# An audit to determine if vitamin b12 supplementation is necessary after sleeve gastrectomy

**DOI:** 10.1186/2193-1801-2-218

**Published:** 2013-05-10

**Authors:** Amar M Eltweri, David J Bowrey, Christopher D Sutton, Lisa Graham, Robert N Williams

**Affiliations:** Department of Surgery, Leicester Royal Infirmary, Level 6 Balmoral Building, Leicester, LE1 5WW UK

**Keywords:** Sleeve gastrectomy, Vitamin B12 deficiency, Anaemia OR sleeve gastrectomy, Cyanocobalamin deficiency OR sleeve gastrectomy, Anaemia

## Abstract

**Abstract:**

Sleeve gastrectomy has increased in popularity over the last five years and it is likely to supersede gastric banding. Nevertheless, it is unclear whether vitamin B12 supplementation is required after surgery. The aim of this short report is to identify any vitamin B12 deficiency and highlight the necessity of post laparoscopic sleeve gastrectomy vitamin B12 monitoring.

**Patients and methods:**

A review of 66 patients underwent LSG in our institution. 25 patients were excluded as they had no postoperative vitamin B12 screening. 41 patients were included as screened for vitamin B12 and other micronutrients including selenium, serum folate, ferritin, iron, zinc, copper, magnesium and vitamin D.

**Result:**

There were 5 male (12%) and 36 females (88%), 8/41 patients (20%) had Vitamin B12 deficiency, none of them developed macrocytic anaemia. 17/21 (81%) patient were vitamin D deficient and 9/21 (43%) exhibited low selenium.

**Conclusion:**

In this small group, a 20% prevalence of vitamin B12 was identified. As a consequence vitamin B12 monitoring and supplementation will be a standard of care in the early postoperative period after LSG at this institution.

## Introduction

Over the last 5 years there has been increasing usage of LSG, both as part of a multistage operation for obese patients and as a definite standalone weight loss procedure (Slotman 2010).

B12 deficiency is a recognised consequence of GBP, and nutrient supplementation has been recommended (Ziegler et al. 2009). There is a paucity of data about the requirement for B12 supplementation after sleeve gastrectomy, although micronutrient deficiency can occur after partial gastrectomy for cancer (Frezza 2007).

As the popularity of sleeve gastrectomy increases, the significance for any concomitant micronutrient deficiency will also increase. The Vitamin B12 screening was not performed routinely for patients undergoing sleeve gastrectomy in our institution. In view of this, we have conducted this retrospective study to identify the early effects of LSG on vitamin B12 absorption and determine the requirement for early monitoring and treatment of cyanocobalamin deficiency.

## Patients and methods

This retrospective study was conducted on 41 morbidly obese patients had undergone LSG at Leicester Royal Infirmary Hospital (England, United Kingdom), between August 2009 until May 2012, 66 patients underwent Laparoscopic sleeve gastrectomy, within this time period. However 25 patients had no vitamin B12 screening post-surgery, and hence were excluded. All Data was inserted in an Excel spread sheet (Microsoft, Redmond, Washington – United States) for statistical analysis. Thirty six females and 5 males were included in this study with a median age 47 years (range 28-62). All patients had a laparoscopic sleeve gastrectomy performed, as a first line weight loss procedure and advised to take 5mg of iron supplements (spatone) once daily, calcium + vitamin D (cacti D3) twice daily and multivitamin (centrum forte) once daily, the later contains 20 microgram vitamin b12, selenium 55 microgram and 0.6 mg of folate as well as another vitamins and minerals. The included 41 patients underwent postoperative laboratory screening for vitamin B12, selenium, serum folate, ferritin, iron, zinc, copper and magnesium, the following tests were used for analysis of the samples: full blood count (ADVIA® 2120 Hematology Siemens Healthcare System), Vitamin B12, Folate, Ferritin (ADVIA® Centaur R System), Iron (ADVIA® 20400 Siemens Healthcare System), Zinc (Flame Atomic Absorption Analytical Methods) and Selenium (Atomic Absorption Analytical Methods).

## Results

Of the 41 patients who underwent LSG at our institution; 20% of these patients showed evidence of Vitamin B12 deficiency, no patient had a hospital stay of more than 5 days or required hospital readmission. Table 1 summarises the nutritional profile of these patients after surgery.

**Table 1:** **Postoperatively laboratory nutritional screen in the study population**

Biochemistry test	Reference values	Low	Normal	High
**Hb**	Male (13-18 g/dl)	0/5 (0%)	5/5 (100%)	0/5 (0%)
	Female(11.5- 16.5 g/dl)	2/35 (6%)	33/35 (94%)	0/35 (0%)
**MCV**	(80-99fl)	2/40 (5%)	38/40 (95%)	0/40 (0%)
**Magnesium**	(0.7-1.0 mmol/l)	0/19 (0%)	19/19 (100%)	0/19 (0%)
**Ferritin**	(10-420 μg/l)	3/37 (8%)	34/37 (92%)	0/37 (0%)
**Folate**	(2.7-17.3 μg/l)	1/35 (3%)	27/35 (77%)	7/35 (20%)
**Vitamin B12**	(220-700 ng/l)	8/41 (20%)	32/41 (78%)	1/41 (2%)
**Zinc**	(8.4-23.0 μmol/l)	0/20 (0%)	20/20 (100%)	0/20 (0%)
**Copper**	(13-24 μmol/l)	0/16 (0%)	15/16 (94%)	1/16 (6%)
**Selenium**	(0.80-2.0 μmol/l)	9/21 (43%)	12/21 (57%)	0/21 (0%)
**Vitamin D**	(50-150nmol/l)	17/21 (81%)	4/21 (19%)	0/21 (0%)
**Albumin**	(32-50 g/l)	0/41 (0%)	41/41 (100%)	0/41 (0%)

Deficiency (Low) was defined as the plasma level of the vitamin/trace element being below the reference values, Normal was defined as the plasma level within the reference values range and High was defined as the plasma level greater than the reference values.

## Discussion

This study shows that 20% of patients that underwent laparoscopic sleeve gastrectomy developed vitamin B12 deficiency.

In 2009 Ziegler O. et al, provided recommendations for the prevention and treatment of nutritional deficiencies; they recommended, that patients should be followed up in the first year following sleeve gastrectomy for evaluation of vitamin B12 deficiency (Ziegler et al. 2009).

This is consistent with five previous studies that have reported on postoperative B12 deficiency following sleeve gastrectomy (Table 2). The incidence of postoperative B12 deficiency from these studies ranges from 0-19.6% up to 36 months follow up after surgery.

**Table 2:** **Published articles reporting vitamin B12 deficiency**

Authors		Vitamin B 12	Folate	Zinc	Ferritin	Iron
**Gehrer S. et al** (Gehrer et al. 2010)	Total (n)	50	50	50	-	50
	Deficiency n (%)	18%	22%	34%	NR	18%
	Abnormal level	<150 pmol/l	<6 nmol/l	<11.5 μmol/l		<30 ng/ml
**Toh S.Y. et al** (Toh et al. 2009)	Total (n)	9	5	-	9	9
	Deficiency n (%)	0%	0%	NR	0%	11%
	Abnormal level	<145pmol/l	<7nmol/l		<15μg/l	<9μmol/l
**Aarts E.O. et al** (Aarts et al. 2011)	Total (n)	60	60	-	-	60
	Deficiency n (%)	9%	15%	NR	NR	43%
	Abnormal level	<150pmol/l	<9nmol/l			<9μmol/l
**Hakeam A.H. et al** (Hakeam et al. 2009)	Total (n)	61	61	-	61	61
	Deficiency n (%)	19.6%	6.5%	NR	1.6 %	4.9%
	Abnormal level	<145pmol/l	<776 nmol/l		Male <30μg/l	<8μmol/l
					Female<13μg/l	
**Kehagias I. et al** (Kehagias et al. 2011)	Total (n)	28	28	-	28	28
	Deficiency n (%)	4%	0%	NR	18%	18%
	Abnormal level	<200pg/ml	<1.5ng/ml		<9ng/ml	<50mg

NR= not reported in the study.

The need for postoperative monitoring for nutritional deficiencies following RYGB is well recognised. However, the need for monitoring nutritional deficiency after LSG is less clear. This study and small number of previous studies have highlighted that B12 deficiency is not uncommon following laparoscopic sleeve gastrectomy.

Vitamin B12 and micronutrients deficiency is a common result after GBP and partial gastrectomy for gastric cancer. This risk can be increased by restrictive surgeries if patients have low intake of meat or dairy products. Vitamin B12 usually absorbed from the small bowel at the terminal ileum in the presence of the intrinsic factor which IS secreted by the parietal cells in the body of the stomach. Resection of a significant proportion of the gastric body during LSG may reduce the productioniof intrinsic factor. Vitamin B12 deficiency can be manifested as anaemia (megaloblastic/macrocytic anaemia) or it can be more serious with neurological sequelae including spinal cord degeneration, which if untreated may lead to permanent neurological deficit. However, detecting Vitamin B12 deficiency in early stages can be treated very easy with oral or even parenteral supplements (Ziegler et al. 2009).

Micronutrients monitoring is essential after bariatric surgery including sleeve gastrectomy; in this small study population, there were 43% of patients that had low selenium levels. Such deficiency may cause cardiomyopathy, mood changes, tiredness, depression and anxiety.

The American Association of Clinical Endocrinologist, The Obesity Society and The American Society for Metabolic and Bariatric Surgery has recommended (R120) that there is insufficient evidence to support selenium screening for deficiency and supplementation in bariatric surgery patients (Mechanick et al. 2008). The American society of metabolic and bariatric surgery recommends that patients having sleeve gastrectomy follow the nutritional supplementation guidelines for the Roux-en-Y gastric bypass (Mechanick et al. 2008; University of Missouri Health System).

Data from bariatric services of the University of Missouri reported that normal levels of vitamin B12 is 180-914 pg/ml and the patient can have symptoms if the level fall below 400 pg/ml. (University of Missouri Health System) In this study there were 31/41 patients (76%) had their vitamin b12 level less than 400 pg/ml.

The limitations of this study were; it was a retrospective study, and due to vitamin B12 not routinely checked for patients undergoing sleeve gastrectomy, there were no preoperative values for comparison.

Another limitation here was the lack of information on the nutritional intake of these patients. The strength of this study was the outcome highlighted the requirement of Vitamin B12 monitoring and supplementation as required after laparoscopic sleeve gastrectomy at this institution.

Laparoscopic sleeve gastrectomy is a relatively new and as such there has been little research conducted to identify the risk of mineral and vitamin deficiencies. This study has demonstrated a significant burden of micronutrient deficiency with LSG. We recommend a prospective study with a large cohort looking for micronutrients deficiency pre and post sleeve gastrectomy in bariatric patients.

## In summary

Vitamin B12 deficiency is evident among sleeve gastrectomy patients; this effect might exacerbate preoperative deficiencies. Routine postoperative vitamin B12 monitoring and supplementation if deficient is required following LSG, this will be a standard of care in the early postoperative period after LSG at this institution.
